# Soil strength influences wheat root interactions with soil macropores

**DOI:** 10.1111/pce.13659

**Published:** 2019-10-30

**Authors:** Jonathan A. Atkinson, Malcolm J. Hawkesford, William R. Whalley, Hu Zhou, Sacha J. Mooney

**Affiliations:** ^1^ School of Biosciences University of Nottingham, Sutton Bonington Campus Loughborough LE12 5RD UK; ^2^ Rothamsted Research, West Common Harpenden AL5 2JQ UK; ^3^ State Key Laboratory of Soil and Sustainable Agriculture Institute of Soil Science, Chinese Academy of Sciences Nanjing 210008 P.R. China

**Keywords:** bulk density, macropore, soil compaction, wheat, X‐ray computed tomography

## Abstract

Deep rooting is critical for access to water and nutrients found in subsoil. However, damage to soil structure and the natural increase in soil strength with depth, often impedes root penetration. Evidence suggests that roots use macropores (soil cavities greater than 75 μm) to bypass strong soil layers. If roots have to exploit structures, a key trait conferring deep rooting will be the ability to locate existing pore networks; a trait called trematotropism. In this study, artificial macropores were created in repacked soil columns at bulk densities of 1.6 g cm^−3^ and 1.2 g cm^−3^, representing compact and loose soil. Near isogenic lines of wheat, Rht‐B1a and Rht‐B1c, were planted and root–macropore interactions were visualized and quantified using X‐ray computed tomography. In compact soil, 68.8% of root–macropore interactions resulted in pore colonization, compared with 12.5% in loose soil. Changes in root growth trajectory following pore interaction were also quantified, with 21.0% of roots changing direction (±3°) in loose soil compared with 76.0% in compact soil. These results indicate that colonization of macropores is an important strategy of wheat roots in compacted subsoil. Management practices to reduce subsoil compaction and encourage macropore formation could offer significant advantage in helping wheat roots penetrate deeper into subsoil.

## Abstract 2

Root–macropore interactions were visualized and quantified using X‐ray CT. In compacted soil, 68.8% interactions resulted in pore colonization compared with 12.5% in loose soil. Results indicate that the colonization of macropores is an important strategy of roots to go through compacted subsoil in wheat.

## 1 INTRODUCTION

Wheat yields are often restricted by water availability in the summer months leading to post‐anthesis drought (Foulkes, DeSilva, Gaju, & Carvalho, 2016). In water limited environments, yield gains from access to subsoil water sources have been estimated at an average of 30–40 kg grain ha^−1^ mm^−1^ of subsoil water used (Kirkegaard, Lilley, Howe, & Graham, 2007; Lilley & Kirkegaard, 2008; Manschadi, Christopher, deVoil, & Hammer, 2006). Access to deeper water sources in the subsoil by improved root growth has been suggested as a method to combat these yield losses in water‐limited environments such as wheat growing regions in India and Australia (Richards, 2006; Wasson et al., 2012). In wheat growing regions of the southeast of England, where the soil normally reaches field capacity in winter, the soil can be close to saturation at depths of 0.5 m in the middle of the driest summers (e.g., Dodd, Whalley, Ober, & Parry, 2011; Whalley et al., 2006).

Deep, metabolically cheap roots are viewed as an ideotype for optimum water and nitrogen uptake in most cereals (King et al., 2003; Lynch, 2013; Thorup‐Kristensen, Salmerón Cortasa, & Loges, 2009). Several studies have suggested that deep rooting is related to root angle or growth rate (Christopher et al., 2013; Manschadi, Hammer, Christopher, & deVoil, 2008; Richard et al., 2015; Wasson et al., 2012). However, these phenotypes fail to consider the effect of soil structure and strength on root behaviour, which can have a significant impact on the growth and distribution of plant roots in the soil.

Soil structure is defined as the size, shape, and arrangement of solids and pores, their continuity and their capacity to retain and transmit fluids and organic and inorganic substances (Bronick & Lal, 2005; Lal, 1991). The spaces in between soil particles are defined as pores and can be classified by size. Macropores, the main focus of this study, are commonly regarded as pores larger than 75 μm in diameter (Soil Sciences Glossary Terms Committee, 2008). Pores formed by biological activity such as plant roots or earthworms are termed *biopores* (Kautz et al., 2014).

There have been numerous studies into the effects of pores on root behaviour. Dexter (1986) conducted model experiments to estimate the probability of roots entering a macropore in an impenetrable subsoil after elongation in an aggregated layer and found in a well‐aerated soil; pore location could be described by a probability function, whereas in poorly aerated soil, it was not possible to rule out active growth of roots towards biopores (termed by Dexter as trematotropism).

Lampurlanés and Cantero‐Martínez (2003) found higher root density in subsoil under no‐till than compared with conventional tillage. They speculated roots could follow biopores to deeper soil layers. Biopores are often associated with earthworm abundance or old root channels. Ehlers, Köpke, Hesse, and Böhm (1983) reported that oat roots were able to exploit earthworm channels present in a no‐tillage system. Wheat roots have been observed to grow in pores created by previous crop roots, earthworm channels, or cracks in the compacted subsoil layer (Barraclough & Weir, 1988; Hodgkinson et al., 2017; White & Kirkegaard, 2010). Experiments described by Stirzaker, Passioura, and Wilms (1996) suggested that few, large pores were not a favourable environment for roots, although they did find that barley plants grew better in a network of narrow biopores made by lucerne and ryegrass. Colombi, Braun, Keller, & Walter (2017) reported, from pot experiments, that the early growth of wheat seedlings was correlated with the number of axial roots. In the field, Bai et al. (2019) found that deep rooting appeared to be more likely in wheat with a greater amount of surface roots. This supports the hypothesis that the exploitation of the soil structural pore space by roots is in part related to the probability of a root finding a pore by chance. Although there are many observations that roots can bypass compacted soil by elongating in biopores or other pore networks, it is unclear whether the ability of roots to locate pores is a trait with a biological basis. Continuous macropores have distinct water, gas, and mechanical properties compared with the soil matrix in compacted soil (Kuncoro, Koga, Satta, & Muto, 2014; Lipiec & Hatano, 2003). Therefore, the direction of root growth when encountering a macropore might be affected by multiple factors including soil mechanical impedance, water status, and oxygen stresses (Tracy, Black, Roberts, & Mooney, 2011). Direct study of root–macropore interactions has been technically difficult until the very recent application of X‐ray micro‐computed tomography (CT) in plant and soil sciences, which can visualize and quantify the root–macropore interaction non‐destructively and quickly (Colombi et., 2017; Tracy et al., 2011).

The purpose of this study was to investigate pore location by roots elongating in loose and dense soil. Artificial macropores formed in the subsoil and exploitation of these pores by roots was monitored with X‐ray CT. We used two near isogenic wheat lines, Rht‐B1a (tall) and Rht‐B1c (dwarf), for the experiment. In field conditions, we have previously found that Rht‐B1c tends to have deeper roots than Rht‐B1a (Hodkinson *et al*. 2017; Bai et al., 2019). We sought to confirm if roots can exploit pores to bypass strong layers of soil. We also aimed see if observations with X‐ray CT could actually confirm accounts that roots actively grow towards pores.

## 2 MATERIALS AND METHODS

### 2.1 Soil column design

**Figure 1:**
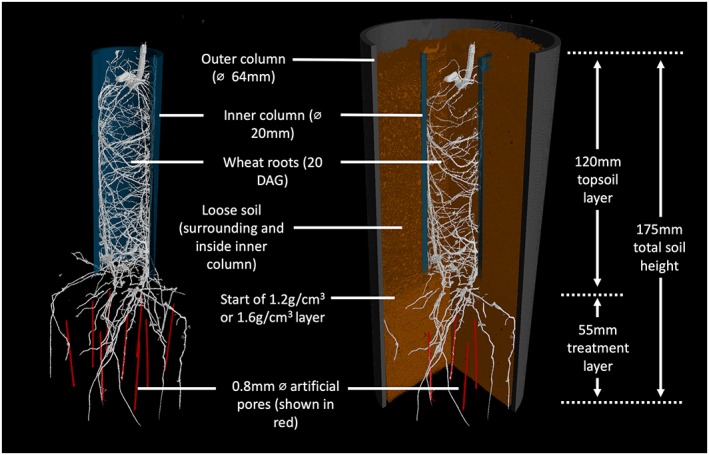
3D reconstruction of the experimental column design with outer column and soil removed (left) and outer column and soil cut away (right). [Colour figure can be viewed at http://wileyonlinelibrary.com]

Field soil was collected from Warren Field at Woburn experimental farm, Bedfordshire, United Kingdom (52°01′11.2″N; 0°35′30.4″W). The field was prepared for cultivation with a mouldboard plough to a depth of 23 cm and is subject to intensive cultivation approaches during establishment of experimental field trails. The soil in the 0–40 cm layer collected for these experiments is a sandy clay loam (Eversley series). Further details of the soil properties can be found in Table 1. This soil was air‐dried and sieved to an aggregate size of <2 mm. Sieved soil was packed to two layers in a polyvinyl chloride column (referred as outer column) with an internal diameter of 64 mm and a height of 170 mm. The bottom 55 mm of the column was packed to simulate subsoil at a bulk density of either 1.2 g cm^−3^ to represent a loose subsoil or 1.6 g cm^−3^ to represent a compacted subsoil. Above the subsoil layer, soil was loosely packed at a bulk density of 1.1 g cm^−3^ to simulate a topsoil layer. In the subsoil layer, nine equally spaced vertical pores (diameter 0.8 mm and length 45 mm) were artificially made by individually inserting and then carefully removing a brass rod into the bottom of the column. A jig was used to ensure identical pore placement and length, and the tip of the brass rod was filed to a cone shape to allow easier passage through the soil. This pore length was selected to leave 10 mm of soil between the top of the artificial pores and the bottom of the topsoil. This allowed roots to grow undisturbed in the upper part of the subsoil layer before interacting with a pore. In the topsoil layer, an inner polyvinyl chloride column (diameter 20 mm and length 120 mm) was vertically placed before filling in the central part of the outer column to restrict root growth to the centre of the soil core. The bottom edge of the inner column was bevelled, allowing it to slightly press into the subsoil layer. The inner column was placed before on top of the subsoil layer before the topsoil layer was added to prevent compaction of the topsoil. A detailed schematic of the mesocosm design is shown in Figure 1. This precise experimental design followed several pilot scale studies to optimize the design including the use of X‐ray imaging to confirm the structural integrity of the artificial macropores.

**Table 1:** Selected properties of the experimental soil

Location		Woburn Expt. Farm Beds.
Grid reference	GB National Grid	SP968364
	Longitude	00:35:30W
	Latitude	52:01:06N
Soil type	SSEW group^a^	Alluvial gley soil
	SSEW series^b^	Eversley
	FAO^a^	Dystric cambisol
Land use		Arable; cereals;beans
Sand (2,000–63 μm)	g g^−1^ dry soil	0.538
Silt (63–2 μm)	g g^−1^ dry soil	0.203
Clay (<2 μm)	g g^−1^ dry soil	0.260
Texture	SSEW class^a^	sandy clay loam
Particle density	g cm^−3^	2.587
Organic matter	g g^−1^ dry soil	0.038
Optimum water content for packing	g g^−1^ dry soil	0.27

a Avery (1980).

b Clayden and Hollis (1984).

SSEW, Soil Survey of England and Wales.

The packed soil columns were saturated slowly by wetting from the base for 48 hr and then allowed to drain freely for a further 48 hr before weighing. Soil water content was maintained throughout the experiment to this weight by weighing and watering every three days. The artificial pores were created after saturating the columns to field capacity to prevent pore collapse during the saturation step.

### 2.2 Plant material and growth conditions

Two wheat near isogenic lines, Rht‐B1a (tall) and Rht‐B1c (dwarf), in a Mercia background were used for this study. Seeds were sieved through a set of calibrated graduated sieves (Scientific Laboratory Supplies Ltd, Hessle, UK) and collected from the 2.8–3.35 mm mesh sizes. Seeds were placed crease‐side down on moistened germination paper and incubated at 4°C for 5 days to synchronize germination. Following the cold treatment, seeds were transferred to a light‐impermeable box for 24 hr to complete germination. The germinated seeds were planted 20 mm below the soil surface within the inner column. Plants were grown in a glasshouse at an average temperature of 22.5°C with supplemental lighting on a 14 hr/10 hr day/night cycle. Soil water content was maintained at field capacity by weighing and watering every three days.

X‐ray μCT scanning was conducted 20 days post‐seed transplantation to maximize the opportunity for root–macropore interactions, despite the probability that some seminal root axes would reach the bottom of the pot in this time. Ten replicates were grown per genotype per soil compaction treatment, giving a total of 40 experimental columns.

### 2.3 CT scanning, image analysis, and data collection

All soil columns were scanned using a v|tome|x M 240 kV X‐ray μCT scanner (GE Sensing & Inspection Technologies GmbH, Wunstorf, Germany) at the Hounsfield Facility at the University of Nottingham, using an electron acceleration energy of 160 kV, current 140 mA, and a resolution of 45 μm. A total of 2,520 projection images were collected during each scan. Reconstruction was performed using Datos|Rec software (GE Sensing and Inspection Technologies GmbH, Wunsdorf, Germany), and 2,000 images were collected for each sample. Considering the time needed for the scanning (44 min), cold treatment, planting, and X‐ray μCT scanning were staggered using a random block design over 6 days to ensure plants were at the same growth stage at the time of scanning.

Image visualisation, soil pore, and root segmentation was conducted using VG Studio MAX (Volume Graphics GmbH, Heidelberg, Germany). The “Region Growing” tool in VG Studio MAX was used to interactively extract roots and artificial pores from the slices as has been described in Helliwell, Sturrock, Miller, Whalley, and Mooney (2019). As we only required segmentation of the artificial pore network and the unconnected roots, no further pre‐ or post‐image processing steps were required.

**Figure 2:**
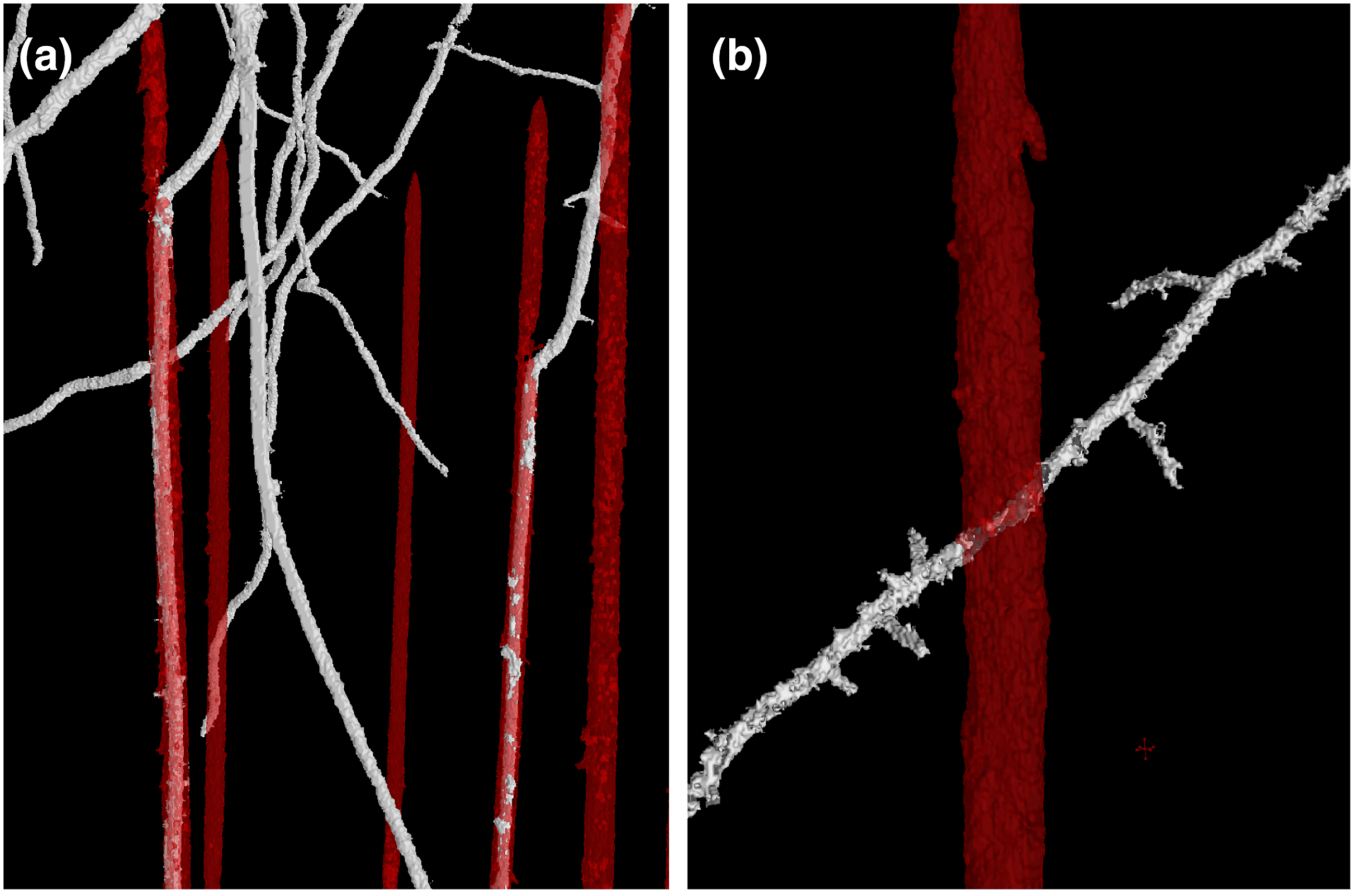
3D reconstruction of segmented root material (white) and artificially generated pores (red). (a) Roots colonizing pores in 1.6 g cm^−3^ soil. (b) A root crossing a pore in 1.2 g cm^−3^ soil [Colour figure can be viewed at http://wileyonlinelibrary.com]

Following segmentation, root–macropore interactions were analysed. A root–pore interaction was defined as a root meeting a pore, with their maximum distance of separation being 1 voxel. Root–macropore interactions were classified as either “crossing” or “colonizing” the pore following an interaction. Colonizing was defined as evidence of the root growing inside the pore for at least >15 mm following interaction, whereas crossing was defined as when a root continued its growth across or away from the pore within 15 mm of initial interaction. Examples of crossing and colonizing are shown in Figure 2.

To further explore the effect of macropores on wheat root growth, the root growth trajectory after each root–macropore interaction was classified as either “change direction” or “no change.” No change in direction was defined as the root continuing at the same growth trajectory as identified at 20 mm pre‐ and post‐pore interaction ±3°, whereas those that exceeded this range were defined as change direction. In cases of pore colonization, by the definition used in this paper, there was a change in growth trajectory. However, in cases where a root crosses a pore, it is possible the root growth trajectory after leaving the pore was different or the same from its initial trajectory before entering the pore. The number of root–macropore interactions was counted and measured.

### 2.4 Penetrometer readings

A further six cores (3 of each treatment, 1.2 and 1.6 g cm^−3^) were prepared for measurement of penetration resistance. Soil cores were prepared by compacting the soil in four separate layers at a pressure of 20 kPa in stainless steel rings of an approximate diameter of 40 mm and height of 36 mm. A needle penetrometer with a cone base diameter of 2 mm and a cone angel of 60° was pushed into the soil core at a speed of 60 mm min^−1^ with an Instron 5940 series load frame fitted with a 100N load. In addition, we investigated the effect of artificial macropores on the strength of the surrounding soils. A vertical hole was made in the soil core using a stainless steel 2 mm drill bit, and the distance between the edge of the hole and the edge of the cone was be measured with a set of callipers. Following the penetrometer measurements, the soil samples were oven dried for 24 hr at 105°C to confirm soil dry bulk density and water content.

### 2.5 Statistical analysis

Statistical analysis was performed using Genstat 19th Edition (VSNI, Hemel Hempstead, UK). The Shapiro–Wilk test was used to check data normality before performing analysis of variance as a randomized block design experiment and plotted the mean data together with the least significant difference for *p* = .05.

## 3 RESULTS

### 3.1 Total root–macropore interactions in the subsoil

The mean number of root–macropore interactions per column was 1.78 ranging between 0 and 7, with no significant difference between genotypes (*p* = .42) or soil treatments (*p* = .84).

### 3.2 Wheat roots colonize and cross macropores

**Figure 3:**
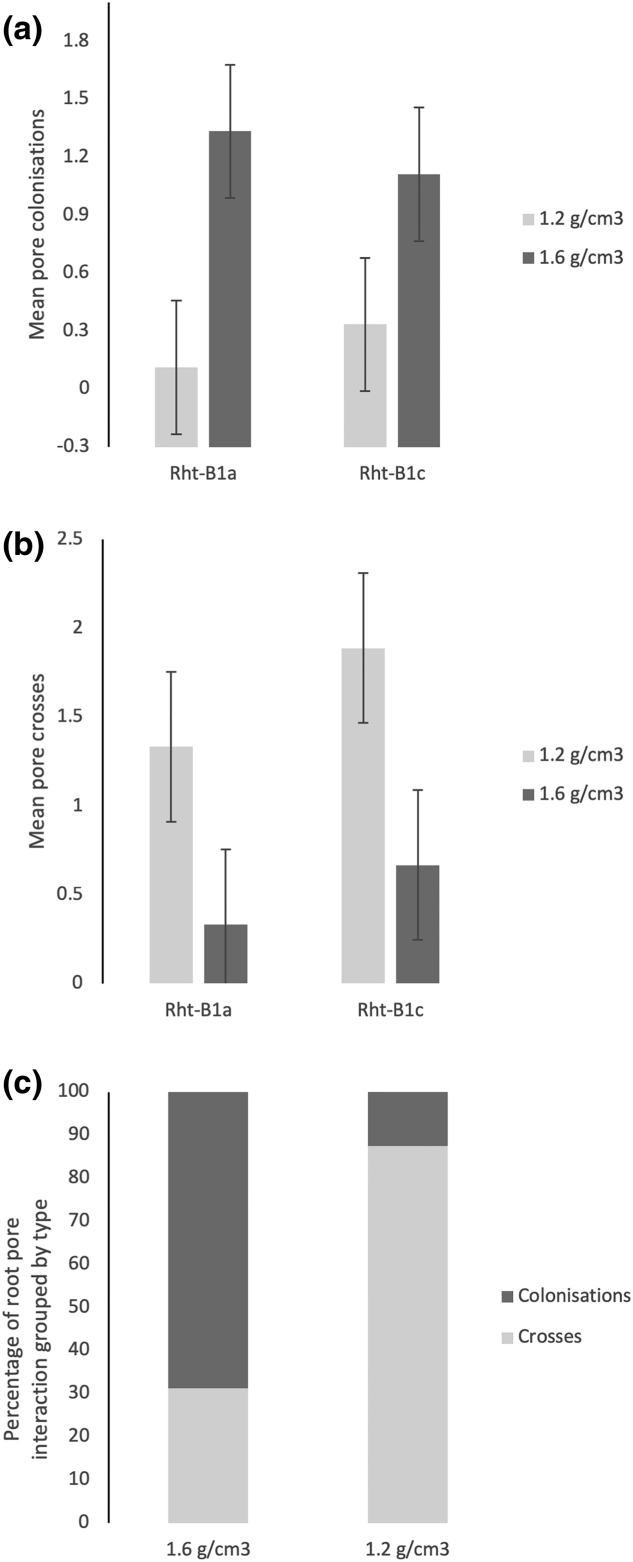
Root–pore interaction data. (a) Mean number pore colonizations. (b) Mean number of pore crosses. (c) Percentage of colonizations/crosses for combined genotype data. Error bars = LSD for *p* = .05

Root response to macropores, that is, colonizing or crossing, showed no significant differences between genotypes for either compacted or loose soil (Figure 3a,b). However, compaction significantly affected root response to macropores for both genotypes (Figure 3a,b). There were significantly more colonizations in the compacted soil than in the loose soil (*p* < .01, Figure 3a). For the Rht‐B1a, 80.0% of root–macropore interactions resulted in colonization compared with 62.5% for the Rht‐B1c. No roots were observed exiting a pore following colonization. In contrast to this, significantly more root crossing a pore was found in non‐compacted soil than in the compacted soil (*p* = .011, Figure 3b), with only 7.7% and 15% root–macropore interactions resulting in colonization for the Rht‐B1a and Rht‐B1c, respectively. The root–macropore interaction data for both genotypes were combined for further analysis because no significant difference was found between genotypes. For the combined data, 68.8% of root–macropore interactions resulted in pore colonization in the compacted soil, whereas only 12.5% resulted in colonization in the loose soil (Figure 3c).

### 3.3 Wheat root growth trajectory after root‐macropore interaction

**Figure 4:**
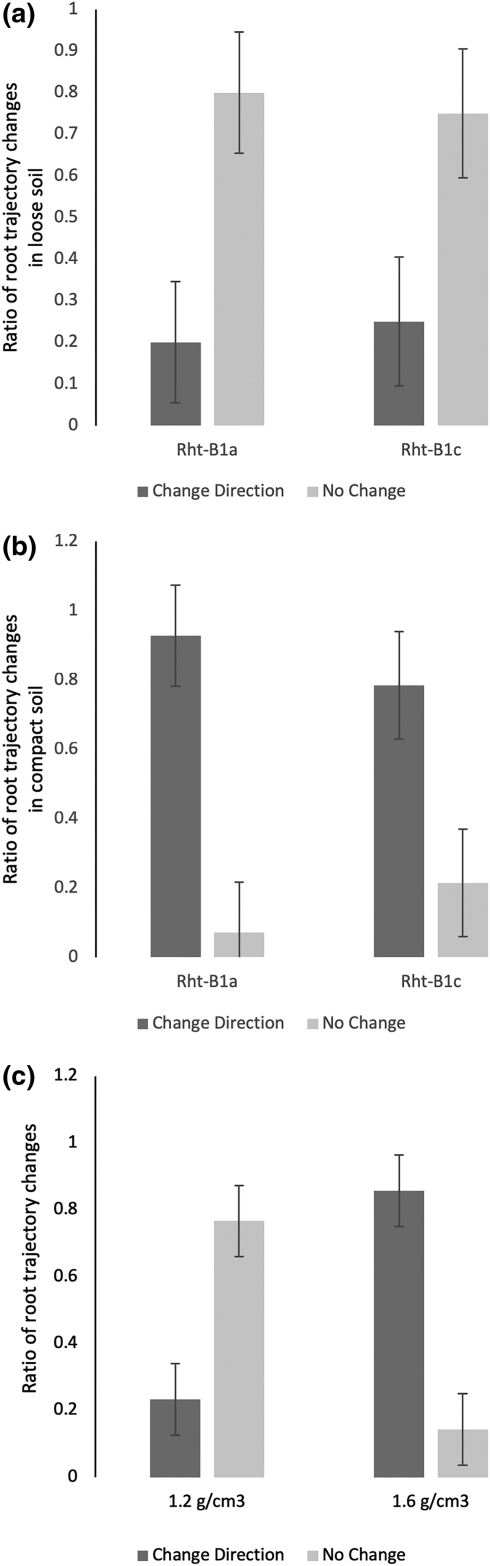
Root trajectory response following pore interaction. (a) Ratio of root response type for each genotype in loose soil (1.2 g cm^−3^). (b) Ratio of root response type for each genotype in compact soil (1.6 g cm^−3^). (c) Ratio of root response type using combined genotype data. Error bars = LSD for *p* = .05

No significant differences in root growth trajectory were found between genotypes in either loose (*p* = .257, Figure 4a) or compacted soil (*p* = .750, Figure 4b). In the loose soil, 20% of Rht‐B1a and 25% of Rht‐B1c roots changed direction following pore interaction. In the compacted soil, root–macropore interactions resulted in a lower percentage of trajectory changes in Rht‐B1c roots (78.6%) compared with Rht‐B1a (92.9%), but this difference was not significant (*p* = .406). The root growth trajectory data of the two genotypes were also combined. For the combined data, significant differences between root direction changes between soil treatments were found (*p* < .001). In compact soil, 76.0% of roots changed direction after root–macropore interactions, whereas only 21.0% changed direction in the loose soil (Figure 4c).

## 4 DISCUSSION

Our data shows that in compacted soil, roots are able to exploit pores to bypass layers of strong soil. Furthermore, imaging showed roots appear to modify their direction of growth to intercept pores. We found that most wheat roots colonized macropores in the compacted subsoil used in our experiment. This is consistent with field studies reporting that at depth (below 0.6 m), wheat roots are predominantly found in macropores (Hodgkinson et al., 2017; White & Kirkegaard, 2010). A study by White and Kirkegaard (2010) found that approximately 50% of root material was found in large pores or cracks at a depth of 0.3 m, increasing to 100% of the root material found in pores below 1 m. In the loose soil, roots did not tend to follow pores but grew across them without any deflection.

Colombi et al. (2017) found wheat roots predominantly crossed macropores without deflection at 1.6 g cm^−3^ but in their soil, this corresponded to a relatively low penetration resistance of approximate 1 MPa, which should not greatly impede root elongation (Yapa, Fritton, & Willatt, 1988). In a similar recent study in Barley that also utilized X‐ray CT, it was possible to directly observe roots leaving 1 mm diameter artificial pores (Pfeifer, Kirchgessner, & Walter, 2014), often pushing from the opposite pore wall to exert the required force to break through. Here, the penetration resistance was 1.4 MPa, and although it was greater than the 1 MPa used by Colombi et al. (2017), it is still low enough for roots to elongate by deforming soil (Bengough & Mullins, 1991). In the present study, penetration resistance was 2.9 MPa in the compact treatment (Table 2), explaining the roots inability to leave the artificial pores. Furthermore, the elongation of roots is particularly sensitive to axial pressure, while somewhat insensitive to radial pressure (Bengough, 2012). This observation explains why roots might preferentially exploit existing pore networks, even if they are smaller than the diameter of the root. In the loose soil, the axial pressure is not high, and roots can proliferate without being influenced pore networks (Figure 2).

**Table 2:** Mean penetrometer resistance readings of the loose 1.2 g cm^−3^ and compact 1.6 g cm^−3^ soil layers at the bottom of the experimental columns

Packed bulk density (g cm^−3^)	Moisture content (g g^−1^)	SD	Measured bulk density (g cm^−3^)	SD	Penetrometer resistance (MPa)	SD
1.2	0.246	0.029	1.196	0.0106	1.088	0.204
1.6	0.253	0.004	1.567	0.0364	2.876	0.1325

*Note*. Penetrometer details can be found in Section 2.

Abbreviation: SD, standard deviation.

**Figure 5:**
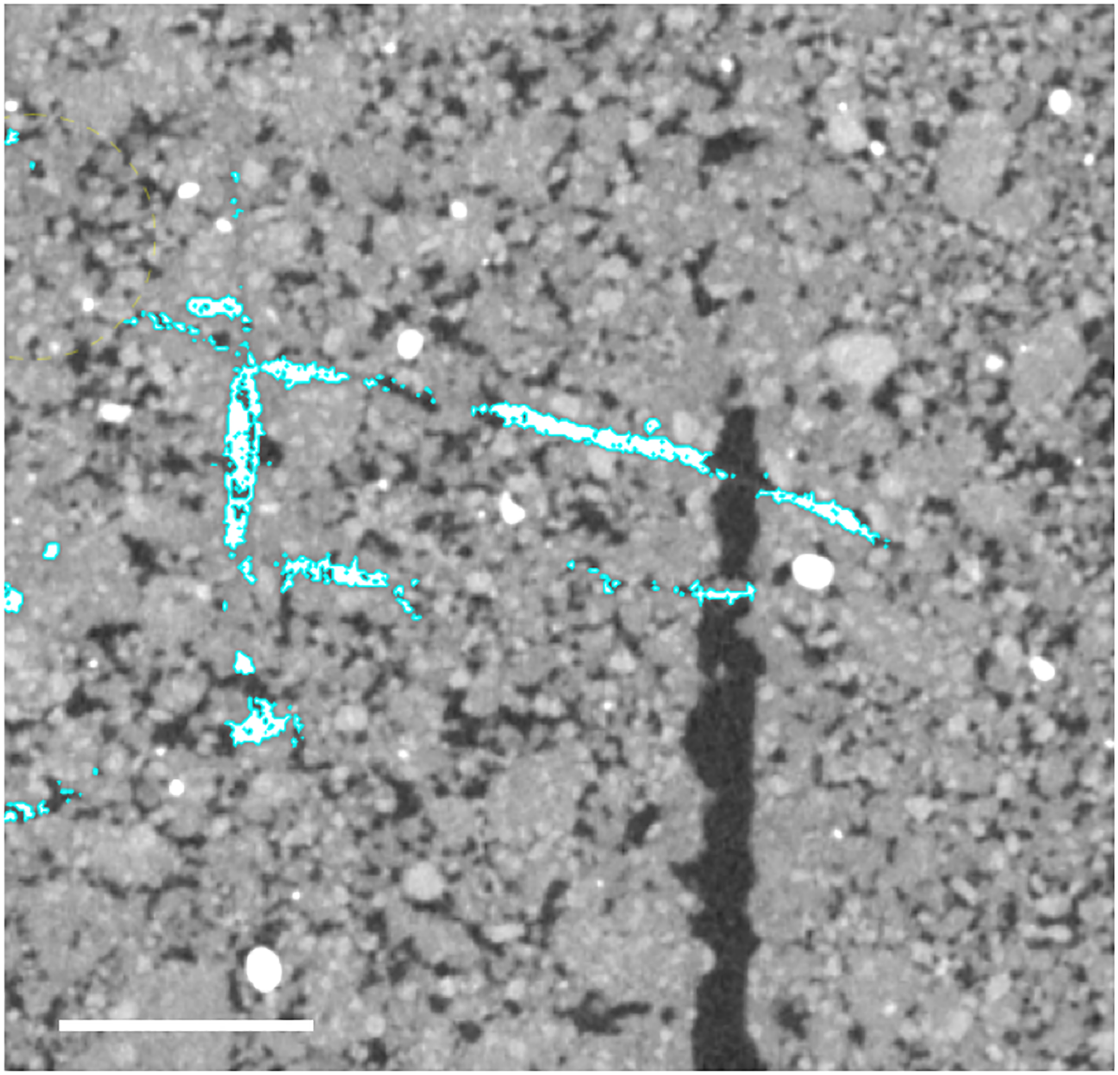
Example ZX 2D X‐ray computer tomography image of segmented roots (white) crossing an artificial pore (black) without changing growth trajectory. Scale bar = 5 mm [Colour figure can be viewed at http://wileyonlinelibrary.com]

In our loose subsoil, root growth in wheat was largely unaffected by the presence of macropores. Of the observed pore interactions in the subsoil with a bulk density of 1.2 g cm^−3^, 87.5% resulted in roots crossing the pore with 76.7% not changing growth trajectory, as illustrated in Figure 5. This suggests that the predetermined root angle is overriding the thigmotropic and trematopic response of the root to the soil matrix and macropore in determining elongation and growth.

However, in the field, it is important to realize that subsoils are almost always strong because of the effects of the weight of the soil, termed the overburden pressure. The effects of overburden pressure on penetration resistance are well‐understood by civil engineers, who mainly use penetrometer measurements to infer material constants of soil. To do this effectively, they need to account for the effect of depth. Gao, Whalley, Tian, Liu, and Ren (2016) have shown that a relatively simple model for penetrometer resistance can be used to describe the effects of bulk density, soil drying, and depth. The relevant point to this study is that increased pressure with depth (due to the weight of soil above) also increases penetration resistance, even if the soil density is the same. The consequence of this is that at relatively shallow depths, for example 0.5 m, penetration resistance can exceed 2.5 MPa, the value at which the elongation of roots by soil deformation is severely restricted (Bengough & Mullins, 1991). Previous measurements in the field from which the experimental soil was collected reported penetration resistance increasing dramatically with depth before any soil drying by roots had occurred (Hodgkinson et al., 2017). Thus, the loose subsoil used in this experiment represents the top soil in the field, and the compact subsoil represents subsoils below 0.5 m (Table 2).

**Figure 6:**
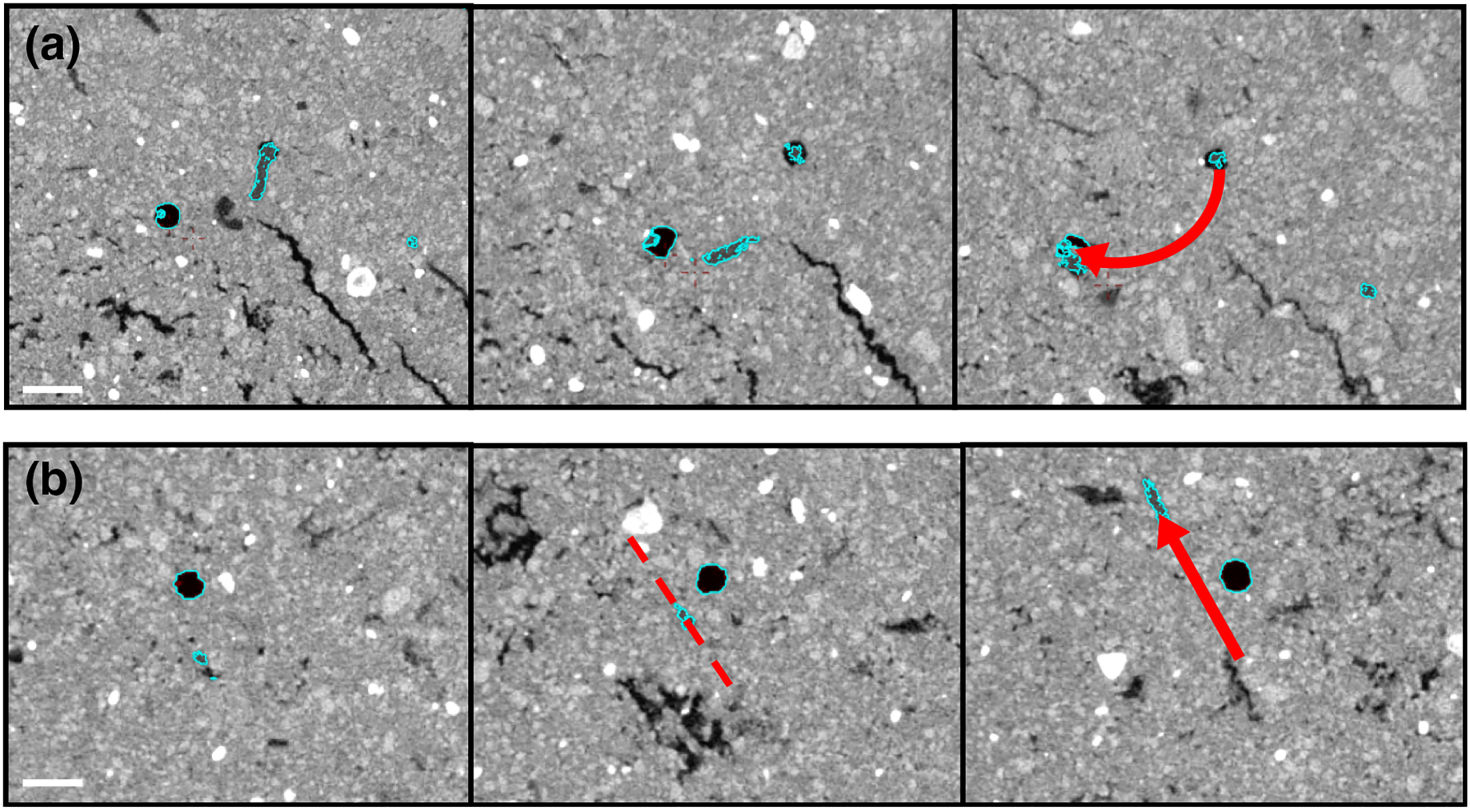
Example XY 2D X‐ray computer tomography images travelling down the column showing (a) root growth towards a pore and (b) root growth past a pore. The pore is shown in black, the root is highlighted in blue, and the growth path is shown by the red arrow. Scale bars = 2 mm [Colour figure can be viewed at http://wileyonlinelibrary.com]

**Figure 7:**
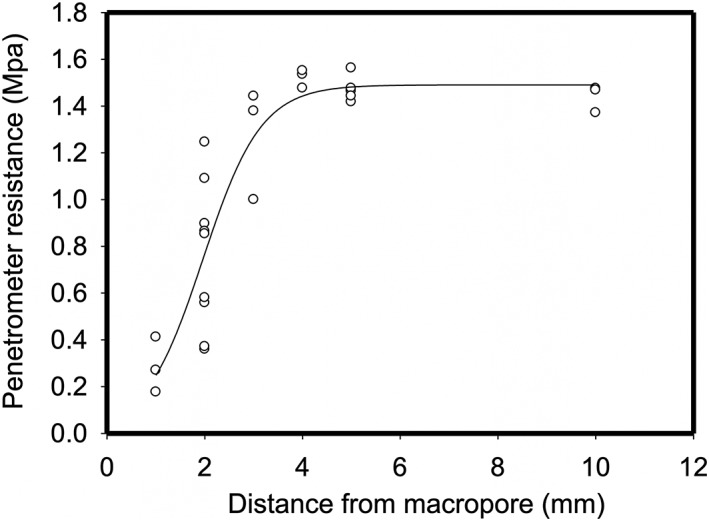
The penetrometer reistance as a function of its distane from a 2 mm hole.

There has been much speculation with respect to the preferential growth of roots towards macropores in compacted soils (Colombi et al., 2017; Pfeifer et al., 2014; Stirzaker et al., 1996). Stirzaker et al. (1996) explained this phenomenon as roots simply following the path of least resistance towards small weaknesses in the soil preceding a larger pore. However, as noted in Pfeifer et al. (2014), in experiments where artificial pores are created by inserting a rod (such as this study), the soil bulk density would be expected to increase around the pore. Currently, it is thought that oxytropism is one possible explanation, particularly at higher bulk densities where oxygen levels might be significantly higher in and around a macropore than in the bulk soil (Colombi & Walter, 2017; Pfeifer et al., 2014). In this study, evidence of this phenomenon can be seen in both Figure 2, where two roots appear to change the direction of elongation when they are near a pore, and also in Figure 6A. However, as illustrated by figure 6B, this observation was not consistent even within the same experimental column. Although it is possible that gradients in oxygen are responsible for preferential root growth towards micropores, another potential explanation is a reduction in penetration resistance in the vicinity of a pore. Figure 7 shows measurements of penetration resistance at different distances from a 2 mm hole made in a soil core. Penetration resistance decreases near the hole because of a reduction in the radial confining pressure. It seems reasonable to speculate that this results in a passive redirection of elongation. When a root meets a pore, the new direction of root growth direction depends on the strength of the soil and the penetration force of root, which are influenced by a variety of factors including plant species, root type/diameter, soil type, pore wall properties, pore age, and soil water content (Dexter, 1986a, 1986b, 1986c).

In field experiments, we have observed that Rht‐B1c is deeper rooting in comparison with Rht‐B1a (Bai et al., 2019; Hodkinson *et al*. 2017). It was assumed that the deeper rooting of Rht‐B1c in the field was related to increased branching, leading to a greater number of roots locating pores. However, under laboratory conditions, we were not able to support this hypothesis. This could be due to the limiting diameter of the soil columns used here, which would affect root architecture and branching. Future effort could focus on the use of X‐ray CT to image soil monoliths extracted from the field. Nevertheless, in this paper, we have highlighted how soil strength and structure interact to determine the distribution of roots.

## 5 CONCLUSIONS

In this study, we investigated the root–macropore interactions in compacted and loose subsoils. Two wheat near isogenic lines were studied, but no significant differences were identified in terms of their response to the macropores in the subsoil. Roots tended to colonize pores in compacted subsoil and change root growth direction, whereas in the loose subsoil, most roots crossed the macropores and did not change growth direction. This suggests a switch in the dominating mechanism in determining root proliferation occurs between 1.2 and 1.6 g cm^−3^ soil bulk density or 1.1 and 2.9 MPa penetration resistance. The precise bulk density and penetration resistance this occurs at, across a range of soil types, is subject for future study. Although pore location by roots has been linked to oxygen gradients, we suggest an alternative mechanism here related to a diminishing root impedance in the soil around pores.

## FUNDING INFORMATION

This work was supported by the Biotechnology and Biological Sciences Research Council Designing Future Wheat Cross‐Institute Strategic Programme [Grant BB/P016855/1] to J.A.A., Z.H., S.J.M., M.J.H., and W.R.W., and the University of Nottingham Future Food Beacon of Excellence funding to J.A.A.
